# Participation of Children with Disabilities in Taiwan: The Gap between Independence and Frequency

**DOI:** 10.1371/journal.pone.0126693

**Published:** 2015-05-11

**Authors:** Ai-Wen Hwang, Chia-Feng Yen, Tsan-Hon Liou, Rune J. Simeonsson, Wen-Chou Chi, Donald J. Lollar, Hua-Fang Liao, Lin-Ju Kang, Ting-Fang Wu, Sue-Wen Teng, Wen-Ta Chiu

**Affiliations:** 1 Graduate Institute of Early Intervention, College of Medicine, Chang Gung University, Tao-Yuan, Taiwan; 2 Department of Public Health, Tzu Chi University, Hualien, Taiwan; 3 Graduate Institute of Injury Prevention and Control, Taipei Medical University, Taipei, Taiwan; 4 Department of Physical Medicine and Rehabilitation, Shuang Ho Hospital, Taipei Medical University, Taipei, Taiwan; 5 School Psychology Program, University of North Carolina at Chapel Hill, Chapel Hill, NC, United States of America; 6 School of Education and Communication, Jönköping University, Jönköping, Sweden; 7 School of Occupational Therapy, Chung Shan Medical University, Taichung, Taiwan; 8 Department of Public Health and Preventive Medicine, Oregon Health & Science University, Portland, Oregon, United States of America; 9 The School and Graduate Institute of Physical Therapy, College of Medicine, National Taiwan University, Taipei, Taiwan; 10 Graduate Institute of Rehabilitation Counseling, National Taiwan Normal University, Taipei, Taiwan; 11 Ministry of Health and Welfare, Taipei, Taiwan; Merced, UNITED STATES

## Abstract

**Background:**

Independence and frequency are two distinct dimensions of participation in daily life. The gap between independence and frequency may reflect the role of the environment on participation, but this distinction has not been fully explored.

**Methods:**

A total of 18,119 parents or primary caregivers of children with disabilities aged 6.0-17.9 years were interviewed in a cross-sectional nationwide survey with the Functioning Scale of the Disability Evaluation System - Child version (FUNDES-Child). A section consisting of 20 items measured the children’s daily participation in 4 environmental settings: home, neighborhood/community, school, and home/community. Higher independence and frequency restriction scores indicated greater limitation of participation in daily activities. Scores for independence, frequency and independence-frequency gaps were examined across ages along with trend analysis. ANOVA was used to compare the gaps across settings and diagnoses for children with mild levels of severity of impairment.

**Findings:**

A negative independence-frequency gap (restriction of frequency was greater than that of independence) was found for children with mild to severe levels of impairment. A positive gap (restriction of independence was greater than that of frequency) was found for children with profound levels of severity. The gaps became wider with age in most settings of children with mild impairment and different diagnoses. Widest negative gaps were found for the neighborhood/community settings than for the other three settings for children with mild to severe impairment.

**Conclusions:**

Children’s participation and independence-frequency gaps depend not only on the severity of their impairments or diagnoses, but also on their age, the setting and the support provided by their environment. In Taiwan, more frequency restrictions than ability restrictions were found for children with mild to moderate severity, especially in the neighborhood/community setting, and increased with age. Further identification of environmental opportunities that positively impact frequency of participation is needed.

## Introduction

Developmental disabilities have been defined as a collection of chronic conditions in childhood and are manifested in cognitive, physical (e.g., motor, sensory), speech, language, or psychological impairments during the developmental period (birth up to 22 years) [1–3]. The launch of the International Classification of Functioning, Disability and Health: Children and Youth Version (ICF-CY) covers the important developmental period of childhood encompassing children’s disabilities and limited functioning of body functions/structures, activities, and participation. Its universal language and coding system can assist clinicians, educators, researchers, administrators, policy makers and parents to document and measure the important growth, health and developmental characteristics of children and youth [4].

Population-based nationwide surveys for children with developmental disabilities, usually enrolling children aged 11 to 22 years [3,5–9], play an important role in documenting epidemiological data, planning of services, evaluating prevention effectiveness and comparing health information. In Taiwan, the Functioning Scale of the Disability Evaluation System—Child version (FUNDES-Child) protocol was developed in conjunction with the launch of the People with Disabilities Rights Protection Act in 2007 [10]. The FUNDES-Child [11–13] is based on the Child and Family Follow-up Survey (CFFS) [14,15] for children and youth aged 6.0–17.9 years. The FUNDES-Child was developed to identify children with developmental disabilities in a nationwide pediatric population to prepare for the implementation of a new service policy based on the components of the ICF-CY.

The overall prevalence of disability or chronic conditions in children reported in nationwide surveys varies widely. For example, prevalence was estimated to be 1.22% in Taiwan [16], 17% in U.S. [7] and up to 30% in Canada [5]. Varying definitions of developmental disabilities is a major contributor to the variation of disability prevalence across countries. In the earlier definition in Taiwan, developmental disability was attributed mainly to health conditions or impairment of body functions, likely to be lifelong in nature and to result in substantial activity limitations and restrictions in societal participation [17]. In the new Disability Eligibility Determination System in Taiwan, rules [11,18] were adopted in which both impairment of body function and a diagnosis of disability are required to be eligible for services. In the US, diagnoses serve as the criteria for disability [7], whereas in Canada, the definition of developmental disabilities was broadened to cover chronic physical health conditions [5].

However, severity of body function impairments and diagnoses can only partly reflect participation restriction of children in daily activities [5,6,19]. The framework of the ICF-CY describes a dynamic interaction between the components of health conditions, body functions/structures, environmental and personal factors that contribute to children’s participation [4].

Based on the United Nations’ Convention on the Rights of the Child, participation has been described as the child’s right to be respected [20] and is considered an ultimate outcome for rehabilitation services based on the framework of ICF/ICF-CY [4,21]. Participation is known to be context-dependent as it describes the functioning of an individual’s social role. Therefore, settings, such as home, school, and community where children participate in daily activities should be identified in the investigation of children’s participation patterns. Recently, researchers have found that children with developmental disabilities face various environmental barriers that restrict their participation in home, school and community contexts [22–28].

In the ICF model (parent to the ICF-CY), Activity is defined as the execution of a task or action by an individual and Participation is defined by involvement in a life situation [4]. The component of Activity and Participation contains two constructs, “Performance” and “Capacity” [4]. However, participation of children has been defined with varied terms and multiple perspectives across measurement tools [29]. The construct of “Performance” describes what an individual “actually does or does do” in his or her current environment. In other words, “Performance” is viewed as “life experience” and is highly context dependent [4]. On the other hand, the construct of “Capacity” is defined as the full ability of an individual assessed in a standardized environment or uniform environment to neutralize the impact of different environments on the ability of the individual [4]. As such it may define what the child “can do in an ideal environment”, usually presented with the basic ability of developmental domains in children. Therefore, the “Capacity” construct identified in ICF is unsuitable to measure “Participation” because “Participation” refers to involvement in a “life situation” rather than in a “standardized” or “uniform” environment.

There is an array of measurable dimensions under the construct of participation in recently developed measures [29]. To explain the abilities in life situations, some researchers proposed the term “Capability” as a construct to clarify and operationalize the measurable dimensions of children’s “independence” or “range of possible functioning” in participation [29–31]. Morris defines capability as “the child’s predicament in the life they lead, taking into account their capacity and available resources, social and physical environment”[32]. Therefore, capability refers to what the child “can do in real life” in contrast to performance as “does do in real life”. In the FUNDES-Child, “capability” or “independence” describes the children’s abilities expected in life situation or in achieving a task or activities as assessed by a caregiver [11,12,14].

Another measurable dimension of participation used in the FUNDES-Child is “frequency”, referring to the extent to which a specific task or activity was carried out [29,33–35]. In this study, “Independence” refers to what a child “can do in real life” in contrast to “Frequency” which describes what a child “does do in real life”. The FUNDES-Child was designed to capture both independence and frequency of children’s participation in daily life as measured by the perspective of parents or primary caregivers [7–9,11–13,36,37].

The gap between “independence” and “frequency” is presumed to reflect the role of contextual facilitators and barriers on functioning and participation in that the environment often provides the context for the difference in what the child “can do” and “does do” [4,21]. However, there has been no clear evidence to support the associations between the independence-frequency gap and other ICF-CY components (heath conditions, body functions, and contextual factors). Furthermore, children of school age face a transition from preschool to school settings, or from home to community contexts, a process that generates new life experiences. For children with disabilities, functional change across the life span, particularly in participation, is critically important for adaptation and could be reasonably regarded as more important than the emergence of basic developmental skills. This distinction is important because the patterns of participation for varying age groups in population-based surveys should be taken into account when making social service policy to identify children’s needs and potential policy interventions.

Participation patterns in varying settings and across ages provide guidance for parents to describe their children’s needs in daily life. The concept of an independence-frequency gap can help in the development of policies and strategies to meet the needs of the children and their families. The purpose of this study was to explore the patterns of gaps in participation between independence and frequency as a function of severity of body functions and structures, diagnosed health conditions, settings and age.

## Materials and Methods

This study utilized a national disability register based on a cross-sectional design of nationwide data collection in Taiwan. This national disability register provided the database of comprehensive profiles of participation in children aged 6.0–17.9 years. A 3-step data analysis was applied. Step 1 used all enrolled children covering a broad range of children to show the global profile of independence and frequency across age and setting in different severity groups. To control for possible confounders, Step 2 limited the children to the ones who had only one of the most frequent diagnoses to examine the effects of severity, setting and diagnosis on the independence-frequency gaps. Step 3 narrowed from Step 2 to include only children with mild level of impairment because of its large sample size that would provide adequate statistical power for each of the 12 age subgroups for trend analysis of frequency, independence and gaps in different diagnosis.

### The study population

This study sample consisted of parents or caregivers of 18,119 children aged 6.0–17.9 years, who were interviewed between July 2012 and January 2014 following the official launch of the Disability Evaluation System (DES) [11,18,38]. The DES is a three-stage evaluation process: (1) medical examination (body functions/structures), (2) functional assessment (participation and environment), and (3) needs assessment [11,38]. Demographic data and health characteristics of the sample are presented in Table 1. The mean ages for the three data analysis steps were 12.1 (SD = 3.5) years, 12.0 (SD = 3.4) years, and 11.9 (SD = 3.4) years respectively. The numbers of children for each year age group were evenly distributed except for a higher percentage in the 12-year old group. The five largest diagnostic groups were intellectual disability (ID), autistic spectrum disorder (ASD), language delay, cerebral palsy (CP), and hearing impairment. The five diagnostic groups were collapsed into four categories in terms of functional limitations [1–3]: cognitive impairment (ID group), psychological impairment (ASD group), language/hearing impairments (LH group), and physical impairments (CP group) for statistical analysis at Steps 2 and 3 in this study.

**Table 1:** Demographic data for children for the three steps of data enrollment.

Variables	Step 1	Step 2	Step 3
	for all the enrolled children	for children having only one type of the five diagnoses^a^	for children with mild severity having only one type of the five diagnoses^a^
	(*N = 18 119*)	(*N = 13 906*)	(*N = 7 719*)
	N (%)	N (%)	N (%)
Sex, male n (%)	11698 (64.6)	9099 (65.4)	5187 (67.2)
Age band (Years)			
6.0–6.9	1724 (9.5)	1336 (9.6)	719 (9.4)
7.0–7.9	1334 (7.4)	1009 (7.3)	598 (7.7)
8.0–8.9	1383 (7.6)	1061 (7.6)	615 (8.0)
9.0–9.9	1397 (7.7)	1067 (7.7)	605 (7.8)
10.0–10.9	1159 (6.4)	888 (6.4)	518 (6.7)
11.0–11.9	1598 (8.8)	1250 (9.0)	754 (9.8)
12.0–12.9	2036 (11.2)	1622 (11.7)	922 (11.9)
13.0–13.9	1352 (7.5)	1030 (7.4)	554 (7.2)
14.0–14.9	1544 (8.5)	1209 (8.7)	644 (8.3)
15.0–15.9	1749 (9.7)	1363 (9.8)	668 (8.7)
16.0–16.9	1377 (7.6)	1022 (7.3)	549 (7.1)
17.0–17.9	1466 (8.1)	1049 (7.5)	573 (7.4)
Major diagnoses/ICD-9-CM codes^b^			
Intellectual disability/317–319	10310 (56.9)	9252 (66.5)	4966 (64.3)
Autism spectrum disorders/299.01–299.90	4013 (22.1)	3331 (24.0)	2117 (27.4)
Language delay/315.31–315.39, 318.1	801 (4.4)	497 (3.5)	326 (4.2)
Cerebral palsy/343.9,	602 (3.3)	413 (3.0)	105 (1.4)
Hearing impairment/389	448 (2.5)	413 (3.0)	205 (2.7)
Schizophrenia/295.10–295.90	191 (1.1)	0	0
Visual impairment/369.0–369.9	89 (0.5)	0	0
Cerebral vascular accident /431–438	58 (0.3)	0	0
Depression/296	31 (0.2)	0	0
Spinal cord injury/952,806	20 (0.1)	0	0
Impairment severity			
Mild	9561 (52.8)	7719 (55.5)	7719 (100.0)
Moderate	5614 (31.0)	4430 (31.9)	0
Severe	1774 (9.8)	1129 (8.1)	0
Profound	1170 (6.5)	628 (4.5)	0
Occupation			
Student	17308 (95.5)	13520 (97.3)	7582 (98.1)
Employee	33 (0.2)	20 (0.1)	12 (0.2)
Quit job/ drop out of school for health reasons	485 (2.7)	181 (1.3)	45 (0.6)
Quit job/ drop out of school for non-health reasons	141 (0.8)	93 (0.7)	44 (0.6)
Others	152 (0.8)	92 (0.6)	36 (0.5)
Child’s physical health in general			
Excellent	1652 (9.1)	1407 (10.1)	840 (10.9)
Very good	3894 (21.5)	3187 (22.9)	1836 (23.8)
Good	4756 (26.2)	3733 (26.8)	2113 (27.4)
Fair	6300 (34.8)	4665 (33.6)	2521 (32.6)
Poor	1508 (8.3)	913 (6.6)	408 (5.3)
Missing	9 (0.1)	1 (0.0)	1 (0.0)
Child’s emotional health and well-being			
Excellent	738 (4.1)	555 (4.0)	335 (4.3)
Very good	2223 (12.3)	1658 (11.9)	913 (11.8)
Good	3995 (22.0)	3052 (21.9)	1733 (22.5)
Fair	7718 (42.5)	6044 (43.5)	3360 (43.6)
Poor	3434 (19.0)	2594 (18.7)	1376 (17.8)
Missing	11 (0.1)	3 (0.0)	2 (0.0)

^a^ The five types of diagnoses are intellectual disability, autism spectrum disorders, language delay, cerebral palsy, and hearing impairment

^b^ Some children have more than one type of diagnosis at Step 1

### Ethics approval

Ethics approval was obtained from Taipei Medical University- Joint Institutional Review Board. This study utilized the de-identified database of Taiwan Databank of Persons with Disabilities (TDPD). The children were assigned a diagnosis with specific codes of the International Classification of Disease, 9th Revision, Clinical Modification (ICD-9-CM) (http://www.cdc.gov/nchs/icd/icd9cm.htm) to be eligible for the DES.

### Measurement of body function

The severity of body functions/structures was assessed in the first stage of the DES. Relevant ICF/ICF-CY categories for specific diagnoses were coded by physicians trained in using a qualifier scale from 0 to 4 points presenting no problem (0), mild (1), moderate (2), severe (3), and profound (4). A final severity level of body function was assessed on the basis of decision rules [39] for combining levels of severity of individual codes of body functions/structures. In this sample, half of the children were classified at mild levels of severity (Table 1).

### Measurement of participation

Child participation was assessed using the FUNDES-Child Section II that contains 20 items to measure 2 dimensions, frequency and independence of participation [12]. The items from the Independence dimension were translated and modified from the Child and Adolescent Scale of Participation (CASP) [15], one part of the CFFS. The Frequency dimension has been designed by the Taiwan ICF team and added to each item of the FUNDES-Child Section II [11]. Items are scored by the 4 domains describing settings (home with 6 items, neighborhood and community with 4 items, school with 5 items, and home/community living activities [HCLA] with 5 items)[12].

The four domains present categories of activities children would experience in the four settings. Activities in the **home** setting include social, play or leisure activities, chores, self-care activities, communication and moving around at home; **community** setting activities include social, play or leisure activities, structured events, moving around and communicating with others in community; **school** setting activities include educational (academic) activities, social, play or leisure activities, moving around, and using educational material in schools; **HCLA activities** include household tasks, shopping and managing money, managing schedule, using transportation to get around, and work activities and responsibility in home and in transition to community. The following are examples of items for specific settings: “Communicating with other children and adults at home” (home), “Social, play or leisure activities with friends in the neighborhood and community” (neighborhood and community), “Using educational materials and equipment that are available to other children in his or her classroom that have been modified for your child” (school), and “Shopping and managing money” (HCLA) [40].

The FUNDES-Child utilizes a proxy format in which parents or caregivers answer questions about their child’s activities in the previous 6 months. In keeping with the format used in the FUNDES-Adult interview [11,38], flash cards with scoring options were used to assist parents in answering questions.

In the training manual and video of the FUNDES-Child, independence was defined as the chi1d’s current level of ability to participate compared to that of other children of his or her age in the same community. For each item, independence was rated as: 0 (independent), 1 (with supervision/ mild assistance), 2 (with moderate assistance), 3 (with full assistance). Frequency of participation was rated with reference to age as: 0 (the same or more than expected for age), 1 (somewhat less than expected for age), 2 (much less than expected for age), and 3 (never does). A response of not applicable (a child would not be expected to do that activity as peers of the same age and in the same community) was allowed for both dimensions. All items were rated under the condition that children used assistive devices as usual. As each item was on the same ordinal scale with the same anchor points at the extreme end (0–3 points), the two dimensions were comparable based on age-expected independence and frequency.

Items rated as "not applicable” were omitted in the scoring [40]. The mean scores for each of the 4 settings of FUNDES-Child Section II are thus the sum of scores all “applicable” items divided by the number of applicable items and then converted to a 0–100 scale. The certified testers could therefore interpret the scores within the same directional framework (higher scores represented greater restriction).

### Training of FUNDES testers

Certified testers associated with 255 DES hospitals in Taiwan administered the FUNDES-Child by interviewing children’s parents or caregivers [41]. The certified testers were professionals licensed as physical therapists, occupational therapists, speech therapists, social workers, clinical psychologists, counseling psychologists, nurses, audiologists, special education teachers, and vocational evaluators. The training programs for certified interviewers covered the procedures of the DES and regulations (30 minutes), introduction to ICF and ICF-CY (30 mins), introduction to assessment instruments (FUNDES-Adult [60 mins], FUNDES-Child [40 mins]), practice of assessment instruments (200 mins), and the web-based platform for entry and storage of data (30 mins). At the end of each training course, a paper-and-pencil test was administered to certify the attending professionals [41].

### Data collection

Children with disabilities were identified and recruited when they entered the DES system accompanied by his/ her caregiver or parents. The first step was usually a visit with one physician followed by the FUNDES-Child assessment with a certified tester in a room in an authorized hospital. The interview and rating of the FUNDES-Child lasted about 40–60 minutes.

### Data entry and analysis

To ensure data quality, all the assessment records were entered by the officers in hospitals into the TDPD, a nationwide web-based information system, and checked by the officer of the local department of health. The physician and the FUNDES qualified tester who completed the disability assessment report were required to enter their names into the information system. The self-detection mechanism of the information system prevented data errors and missing responses. The data were then exported to statistical packages periodically.

**Fig 1:**
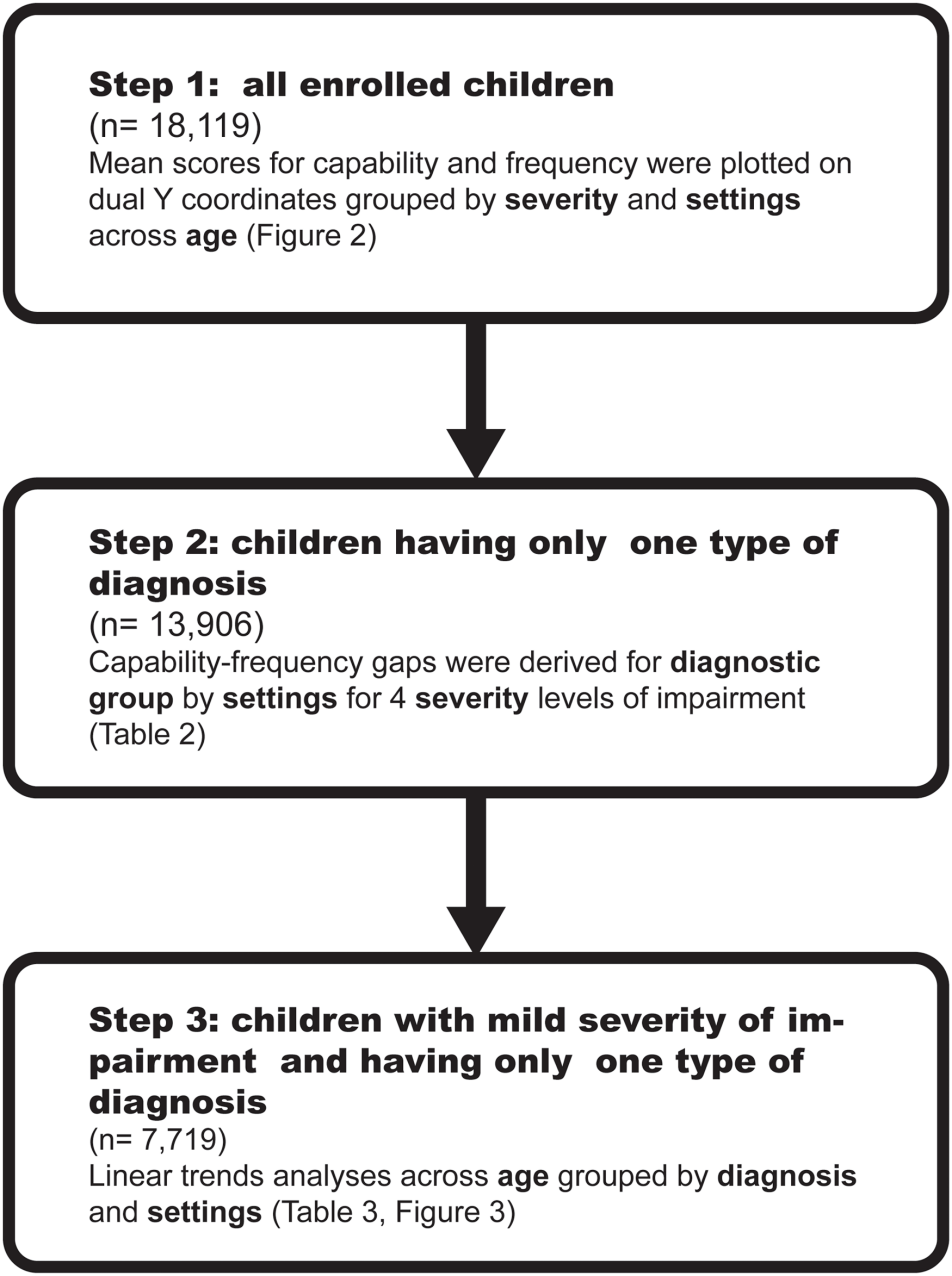
The flow of the three steps of data reduction and analysis.

According to the flow of the three steps of data reduction and analysis (Fig 1), the strategies of analysis are described below.

**Fig 2:**
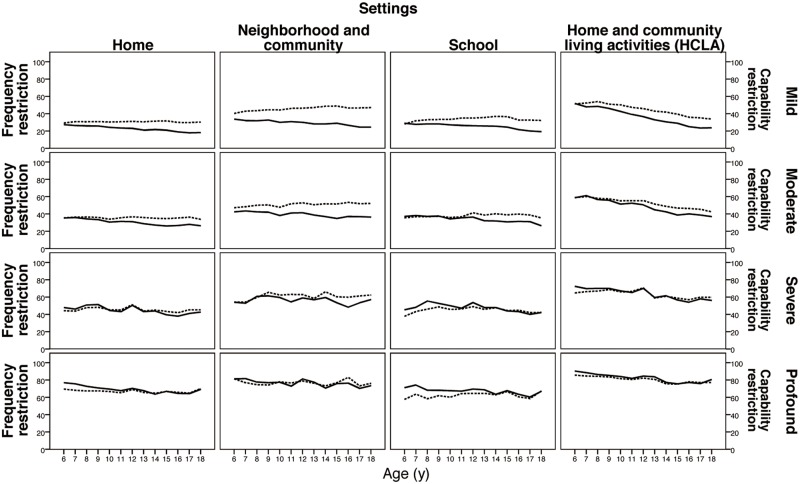
Independence and frequency gap by age, settings and severity levels of impairment for the whole group. Dashed lines are the restriction frequency scores across age; solid lines are restriction independence scores across age.

In Step 1, we included all the enrolled children (n = 18,119) to illustrate the global picture of the changes of independence, frequency and the gaps between them with age as a function as severity and settings. The mean scores for independence and frequency were plotted on dual Y coordinates (from age expected to most restricted based on a 0–100 scale), indicating the discrepancy between independence and frequency grouped by severity and settings across age (Fig 2).

In Step 2, we included the children seen only in one of the more frequent diagnostic groups (n = 13,906) to avoid the issue of comorbidity. The independence-frequency gaps were defined as the scores of independence restriction minus the scores of frequency restriction. If the independence-frequency gap was positive, it meant that restriction of independence was greater than that of frequency (or doing frequency exceeded doing ability); if the gap was negative, it meant that restriction of frequency was greater than that of independence (or doing ability exceeded doing frequency). The gap, discrepancy between independence and frequency, is wider if the values of the positive gap increase or the negative gap values decrease.

The independence-frequency gaps were first derived for 4 severity levels by 4 settings to explore the effects of severity and setting on the gaps. Then, for each severity level, the gaps were analyzed for diagnostic groups by settings to explore the effects of diagnosis and setting on the gaps. Distribution of the severity levels of the majority of the participants (n = 13,906, 76.7%) classified on the basis of the four major diagnostic groups is listed in Table 1. The mean gaps (positive gaps are shown with bold characters and numbers in Table 2) were analyzed across diagnosis/severity with ANOVA, and across setting with repeated measures ANOVA, followed by Scheffé's post hoc analyses (Table 2). Considering the significant discrepancy of sample sizes between diagnostic/severity groups that could contribute to variance inequality, Leven’s test for homogeneity was conducted before ANOVA. Welch ANOVA and Games-Howell post hoc analyses were performed if the data failed to meet the equal variance assumption. The relationship between independence and frequency were analyzed with Pearson’s correlation in four settings for each severity level with alpha set at 0.05 (2-tailed).

**Table 2:** Comparisons of the independence-frequency gaps across setting, severity and diagnosis for children with only one diagnosis of all severity levels in body function.

	Settings^a^					
	Home	Neighborhood and community (NC)	School^b^	HCLA	Significance^c^	Post hoc^d^
Severity levels (n = 13906)						
Mild (n = 7719)	-8.1 (± 14.93)	-16.6 (±22.19)	-8.5 (± 17.35)	-8.2 (± 17.85)	F = 672.61, P<0.001	Home = HCLA = School > NC
Moderate (n = 4430)	-5.7 (± 15.39)	-12.1 (± 20.98)	-4.5 (± 18.13)	-4.4 (± 17.4)	F = 303.17, P<0.001	HCLA = School> Home > NC
Severe (n = 1129)	-1.0 (± 15.12)	-4.9 (± 18.98)	**2.2 (**± **17.46)**	-0.2 (± 16.01)	F = 51.21, P<0.001	**School**>HCLA = Home **>**NC
Profound (n = 628)	**3.3 (± 13.61)**	**1.3 (± 14.88)**	**6.6 (± 17.95)**	**2.5 (± 12.39)**	F = 15.51, P<0.001	**School>Home = HCLA = NC**
Significance	F = 188.70, P<0.001^f^	F = 318.31, P<0.001^f^	F = 232.53, P<0.001^f^	F = 186.87, P<0.001^f^		
Post hoc	**P>**S> Mod> Mil^h^	**P>**S> Mod> Mil^h^	**P**> **S>** Mod> Mil^h^	**P>**S> Mod> Mil^h^		
Mild (n = 7719)						
ID (n = 4966)	-8.7 (± 14.89)	-17.6 (± 22.04)	-8.9 (± 17.37)	-8.3 (± 17.52)	F = 499.75, P<0.001	HCLA = School = Home> NC
ASD (n = 2117)	-6.9 (± 14.50)	-15.1 (± 22.11)	-8.2 (± 17.02)	-7.8 (± 18.14)	F = 142.30, P<0.001	Home = HCLA> School> NC
LH (n = 531)	-7.1 (± 16.07)	-14.0 (± 23.24)	-6.2 (± 17.84)	-7.9 (± 19.05)	F = 31.42, P<0.001	School = Home = HCLA> NC
CP (n = 105)	-4.3 (± 16.88)	-15.2 (± 22.18)	-6.0 (± 16.54)	-5.1 (± 20.04)	F = 14.48, P<0.001	Home = HCLA = School> NC
Significance	F = 10.48, P<0.001^f^	F = 9.88, P<0.001^e^	F = 4.44, P = 0.004^e^	F = 1.67, P = 0.17^e^		
Post hoc	CP = ASD = LH>ID^h^	LH = ASD = CP > ID^g^	CP = LH>ASD = ID^g^	CP = ASD = LH = ID^g^		
Moderate (n = 4430)						
ID (n = 3295)	-6.1 (± 15.37)	-13.0 (± 21.03)	-4.7 (± 18.26)	-4.4 (± 16.82)	F = 271.36, P<0.001	HCLA = School >Home> NC
ASD (n = 805)	-4.6 (± 15.20)	-8.2 (± 19.93)	-4.2 (± 16.80)	-3.4 (± 19.05)	F = 20.62, P<0.001	HCLA = School = Home> NC
LH (n = 205)	-4.8 (± 16.27)	-12.1 (± 21.78)	-4.2 (± 19.01)	-7.1 (± 18.68)	F = 14.23, P<0.001	School = Home = HCLA> NC
CP (n = 125)	-2.6 (± 15.05)	-11.4 (± 22.16)	-1.9 (± 21.02)	-4.1 (± 18.98)	F = 12.79, P<0.001	School = Home = HCLA> NC
Significance	F = 3.84, P = 0.01 ^e^	F = 12.22, P<0.001 ^f^	F = 0.80, P = 0.49 ^f^	F = 2.14, P = 0.95 ^f^		
Post hoc	CP = ASD = LH>ID^g^	ASD>CP = LH = ID^h^	CP = ASD = LH = ID^h^	ASD = CP = ID = LH^h^		
Severe (n = 1129)						
ID (n = 673)	-1.7 (± 16.12)	-5.3 (± 19.70)	**1.7 (± 17.93)**	-0.5 (± 16.64)	F = 29.46, P<0.001	**School** > HCLA = Home **>**NC
ASD (n = 260)	-0.2 (± 13.16)	-3.7 (± 16.67)	**2.1 (± 16.83)**	**1.5 (± 15.43)**	F = 9.48, P<0.001	**School** = **HCLA>** Home >NC
LH (n = 124)	-1.0 (± 13.44)	-7.2 (± 21.98)	**2.7 (± 15.44)**	-2.2 (± 16.01)	F = 9.15, P<0.001	**School** > Home = HCLA**>**NC
CP (n = 72)	**2.6 (± 14.44)**	-1.4 (± 13.16)	**6.2 (± 18.56)**	-2.1 (± 10.50)	F = 5.14, P = 0.002	**School** = **Home>** NC = HCLA
Significance	F = 2.25, P = 0.08 ^f^	F = 2.47, P = 0.06 ^f^	F = 1.38, P = 0.25 ^e^	F = 2.29, P = 0.77 ^e^		
Post hoc	**CP** = ASD = ID = LH^h^	CP = ASD = ID = LH ^h^	**ID = ASD = LH = CP** ^g^	**ASD =** ID = CP = LH^g^		
Profound (n = 628)						
ID (n = 318)	**3.0 (± 14.22)**	**0.9 (± 15.89)**	**6.0 (± 18.53)**	**2.2 (± 12.46)**	F = 6.68, P<0.001	**School**>**Home** = **HCLA**>**NC**
ASD (n = 149)	**1.9 (± 11.58)**	**0.4 (± 13.10)**	**4.9 (± 14.17)**	**3.7 (± 13.42)**	F = 4.55, P = 0.004	**School**> **HCLA** = **Home** = **NC**
LH (n = 50)	**2.0 (± 13.90)**	**2.7 (± 14.36)**	**8.5 (± 20.36)**	**1.0 (± 8.24)**	F = 2.81, P = 0.060	**School** = **NC** = **Home** = **HCLA**
CP (n = 111)	**6.7 (± 13.85)**	**3.3 (± 14.28)**	**10.6 (± 20.00)**	**2.24 (± 12.16)**	F = 8.02, P<0.001	**School** = **Home**> **NC** = **HCLA**
Significance	F = 2.97, P = 0.03 ^e^	F = 0.98, P = 0.40 ^e^	F = 1.88, P = 0.14 ^f^	F = 0.91, P = 0.44 ^f^		
Post hoc	**CP> ID = LH = ASD** ^**g**^	**CP = LH = ID = ASD** ^**h**^	**CP = LH = ID = ASD** ^**h**^	**ASD = CP = ID = LH** ^**h**^		

^a^ Values are mean (±SD) of the gap of the scores between independence and frequency, and the values in bold present positive gaps.

^b^ The domains of school setting are only applicable for children with the occupation of student, therefore the number of participants (n = 13,520) of this domains would be less than the other domains (n = 13,906).

^c^ The values of the gaps were compared across setting using repeated measured ANOVA.

^d^ The post hoc analyses across settings were performed using Scheffé's method.

^e^ The values of the gaps were compared across diagnostic/severity groups using one-way ANOVA.

^f^ The values of the gaps were compared across diagnostic/severity groups using Welch ANOVA because the data fail to meet the equal variance assumption by significant Levene's test for homogeneity of variances

^g^ The post hoc analyses across diagnostic groups were performed across diagnostic groups with Scheffé's method.

^h^ The post hoc analyses across diagnostic/severity groups were performed using Games-Howell post hoc analysis because the data fail to meet the equal variance assumption by significant Levene's test for homogeneity of variances.

HCLA = home and community living activities; ID = Intellectual disability; ASD = Autistic spectrum disorder; LH = language/hearing impairment; CP = Cerebral palsy; Mil = mild; Mod = moderate; S = severe; P = profound

**Fig 3:**
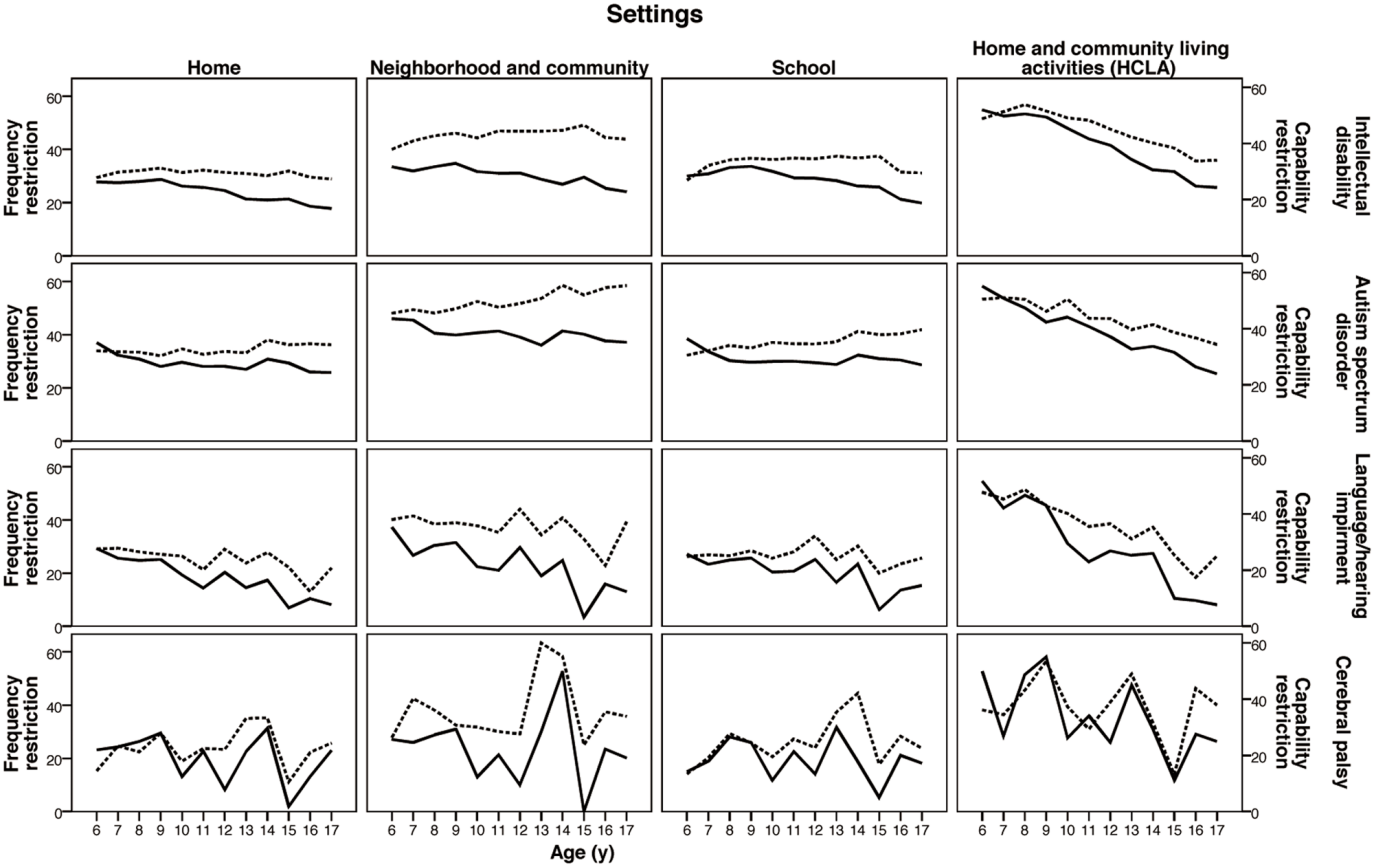
Independence and frequency gap by age, settings and diagnoses for the children with mild severity. Dashed lines are the restriction frequency scores across age; solid lines are restriction independence scores across age.

In Step 3, only the children with mild severity (n = 7,719) were included to examine the nature of the independence-frequency gap as a function of the age by diagnosis/setting interaction with linear trend analysis. Trends were plotted by age on dual Y coordinates, contrasting the discrepancy between independence and frequency (Fig 3). Linear trends analyses with the significance level set at 0.05 (2-tailed) were conducted for independence, frequency, and the gaps across ages (Table 3). The significance of the linear trend was tested by ANOVA for 12 age groups, and the between-groups sum of squares for the effect of age was partitioned into a linear trend and higher order trends. The linear trend component was tested by an F-ratio (the mean square for linear trend/error term). The statistical analyses in Step 2 and Step 3 were performed with SPSS 20.0 (IBM SPSS, Chicago, Illinois, 2013).

**Table 3:** Mean scores of frequency, independence, and gaps between independence and frequency across setting and diagnosis and the trend analyses for children with mild severity in body function.

	Frequency		Independence		Independence-frequency gap ^a^	
	Mean (±SD)	Trend with age	Mean (±SD)	Trend with age	Mean (±SD)	Trend with age
Home						
ID	30.8 (± 17.58)	Up, F = 5.10, P = 0.02	22.1 (± 16.59)	Dn,F = 190.24,P<0.001	-8.7 (± 14.89)	Wider, F = 155.56, P<0.001
ASD	34.3 (± 16.66)	Up, F = 5.10,P = 0.02	27.4 (± 16.52)	Dn, F = 30.41, P<0.001	-6.9 (± 14.50)	Wider, F = 80.53, P<0.001
LH	26.0 (± 17.21)	Dn, F = 8.10, P = 0.01	18.9 (± 16.56)	Dn, F = 43.11, P<0.001	-7.1 (± 16.07)	Wider, F = 11.55, P = 0.001
CP	23.2 (± 18.57)	n.s., F = 0.03, P = 0.87	18.9 (± 17.39)	n.s., F = 2.77, P = 0.10	-4.3 (± 16.88)	Wider, F = 4.19, P = 0.04
Neighborhood and community						
ID	45.4 (± 24.04)	Up, F = 4.44, P = 0.04	27.7 (± 23.3)	Dn, F = 72.75, P<0.001	-17.6 (± 22.04)	Wider, F = 125.91, P<0.001
ASD	52.8 (± 23.08)	Up, F = 35.93, P<0.001	37.8 (± 25.23)	Dn, F = 13.48, P<0.001	-15.1 (± 22.11)	Wider, F = 116.50, P<0.001
LH	37.8 (± 26.08)	n.s, F = 0.82, P = 0.37	23.7 (± 22.7)	Dn, F = 20.97, P<0.001	-14.0 (± 23.24)	Wider, F = 12.20, P = 0.001
CP	36.5 (± 27.25)	n.s, F = 0.53, P = 0.47	21.2 (± 21.7)	Dn, F = 20.97, P<0.001	-15.2 (± 22.18)	Wider, F = 5.42, P = 0.02
School						
ID	34.9 (± 21.03)	n.s, F = 0.91, P = 0.34	26.0 (± 19.36)	Dn, F = 162.63, P<0.001	-8.9 (± 17.37)	Wider, F = 169.50, P<0.001
ASD	37.2 (± 20.89)	Up, F = 23.92, P<0.001	29.0 (± 19.25)	Dn, F = 9.43, P = 0.002	-8.2 (± 17.02)	Wider, F = 92.67, P<0.001
LH	26.9 (± 21.63)	n.s., F = 2.47, P = 0.12	20.7 (± 18.98)	Dn, F = 13.31, P<0.001	-6.2 (± 17.84)	n.s., F = 3.76, P = 0.05
CP	26.0 (± 23.50)	n.s, F = 1.35, P = 0.25	20.0 (± 20.49)	n.s., F = 0.33, P = 0.57	-6.0 (± 16.54)	Wider, F = 5.32, P = 0.02
Home and community living activities						
ID	46.0 (± 22.28)	Dn, F = 437.71, P<0.001	37.7 (± 23.23)	Dn, F = 890.45, P<0.001	-8.3 (± 17.52)	Wider, F = 118.65, P<0.001
ASD	45.7 (± 22.10)	Dn,F = 133.44, P<0.001	37.9 (± 22.24)	Dn, F = 330.79, P<0.001	-7.8 (± 18.14)	Wider, F = 50.81, P<0.001
LH	40.6 (± 23.98)	Dn,F = 64.85, P<0.001	32.7 (± 24.56)	Dn, F = 131.65, P<0.001	-7.9 (± 19.05)	Wider, F = 10.51, P = 0.001
CP	41.6 (± 25.11)	n.s., F = 1.16, P = 0.28	36.5 (± 25.26)	Dn, F = 5.85, P = 0.018	-5.1 (± 20.04)	n.s., F = 3.04, P = 0.09

^a^ Values are showed for the scores of independence minus the scores of frequency

Up = significantly upward trend; Dn = significantly downward trend; ns = non-significant; ID = Intellectual disability (n = 4966); ASD = Autistic spectrum disorders (n = 2117); LH = language/hearing impairment (n = 531); CP = Cerebral palsy (n = 105).

## Results

For Step 1, among the 18,119 children, 1,106 (6%) children had two of the four diagnoses, and only 16 (< 0.1%) children had more than two diagnoses. Among the children diagnosed with an intellectual disability (n = 10,310) (Table 1), 650 children (6%) were also diagnosed with ASD, 264 (3%) with language/hearing impairments, and 154 (1%) children with cerebral palsy. Furthermore, there was significant association between impairment severity and the number of comorbid conditions (Chi-square = 1145.38, P<0.001). Of the 9,561children with mild severity, 7,720 children (81%) had only one diagnosis, while of the 1,170 children with profound severity, 540 children (46%) had two or more diagnoses. Of the 18,119 children, 45% lived in the northern part of Taiwan, which has the highest level of urbanization, 21% in the central part, 27% in the southern part, with 7% in the western part and some remote islands, which have the lowest level of urbanization.

Fig 2 provides the complete picture of the settings, severity level of impairment and the independence-frequency gap by age. A negative gap was found for children with mild levels of impairments for all settings (Fig 2 top row). As the gaps increased with age, the significance of the trends were further examined in Step 3. For children with moderate to profound levels of impairment, the trends varied by setting. A positive gap was found even for children with severe to profound levels of impairments, especially for children aged 6–9 years in home and school settings (Fig 2 lower rows in home and school settings).

The analyses for Step 2 for those children with only one diagnosis revealed a similar pattern to the whole group; that is, the mean gaps were all negative for children with mild to moderate levels of impairment, shifting to positive for children with severe level of impairment in the school setting, and were all positive for children with profound level of impairment (Table 2). Comparing gaps among different severity groups with ANOVA, there were significant differences among the 4 groups (Table 2, first box). Largest positive gaps were found for children with profound level of severity in all the 4 settings with the widest negative gaps for the mild group. Comparing gaps among different diagnostic groups, wider negative gaps were found among children with mild or moderate ID in the home, neighborhood/community, and school settings, and children with mild ASD in the school setting (Table 2, 2^nd^ and 3^rd^ box). A significantly greater positive gap was found only for the profound children with CP in home setting (Table 2, 5^th^ box).

Across the four settings, the widest negative gap was found significantly in the setting of neighborhood/community for the four diagnostic groups of children with mild to severe level of impairments based on the post hoc analysis (Table 2). For children with a profound level of impairment, the largest positive gaps were in the school setting, especially for children with ID and ASD (Table 2, 5^th^ box).

The correlations between independence and performance for the four settings were moderate (*r* = 0.59–0.70, *P*<0.001) for children with mild severity, were moderate to high (*r* = 0.66–0.76, *P*<0.001) for children with moderate severity, were high for both severe (*r* = 0.77–0.84, *P*<0.001) and profound groups (*r* = 0.75–0.84, *P*<0.001).

The results of the Step 3 analysis are shown in Table 3, with mean values of frequency, independence, gap and the corresponding trends with age for children with mild severity and graphically presented in Fig 3. A decreasing trend of independence restriction with age was found for all diagnostic groups except children with CP at home and school settings, indicating that parents perceived improvement of the children’s ability in daily activities. The trends of frequency, however, are more variable than trends for independence across age groups. A review of the trend lines in Table 3 and Fig 3 indicated that the gaps for each setting by diagnostic group were significantly wider with age except for two diagnostic groups by settings (LH in school setting, and CP in HCLA setting).

## Discussion

The results of this large-sample study provide a picture of independence and frequency of participation using a broad distribution of severity and diagnoses of childhood disabilities in Taiwan. About half of the sample represented children with ID (57%) or were mildly impaired in body functions/structures (53%).

For the analysis of Step 2 in this study, widest negative gaps between independence and frequency (indicating potentially more environmental barriers) were found for neighborhood/community settings than the other three settings for children with mild to severe levels of impairment (Table 2, first box), especially for children aged 6–9 years (Fig 2). School and home may be the settings that provide relatively sufficient support for the less severe and younger child to engage in activities. For children with profound levels of impairment, the discrepancy between independence and frequency even changed to positive gaps (indicating hypothesized environmental support) especially in school settings. It appears that children with severe and profound disabilities receive more support in school settings (Table 2, 1st and 4^th^-5^th^ boxes). Previous studies have shown that children with disabilities experience barriers for participation in different settings, especially with regard to physical demands and needs in school. These areas were largely unmet in accommodations of special computer equipment, adapted furniture or accessibility of school buildings [24,42–44]. In this study, however, the children with ID or ASD with mild severity were even less supported than children with physical disability in social participation (Table 2, 2^nd^ boxes).

Social and institutional barriers have been identified as the most significant environmental barriers in studies of children with disabling conditions in Canadian communities [26,45]. Parents reported that their children encountered greater barriers to participate in school and work environments, and the barriers included problems with service, assistance, attitude, and policy. Poorly designed barrier-free community facilities, difficulty getting information about services and programs, the vague process of applying to schools, and negative attitudes were participation challenges faced by children in these studies [26,45]. The attitudes and abilities of teachers to modify courses and instruction were also important influential factors on the extent of children’s participation [26,45].

Further statistical analyses in Step 3 indicated that the independence restriction to participate in daily activities decreased significantly with age among the children with mild severity, except for children with CP in home and school settings. The trend of frequency with age, however, varied across the impairment by diagnosis clusters (Table 3). The trend of gaps showed that the mean frequency restriction of participation was higher than mean independence across settings and diagnoses and the mean independence-frequency gap became progressively wider with age across settings (Fig 3). This may suggest that children with mild severity have fewer opportunities to do tasks than they are capable of doing as they transition to adulthood.

It should be noted that for children with mild severity of impairment, data for independence and frequency for children with ID and ASD were relatively more restricted than for children with CP or LH (Table 3). The negative gap was also more obvious in children with mild rather than severe impairment (Table 2). This may be due to the social nature of participation and the fact that the children with ID or with mild impairment face more environmental barriers (suggested by negative gaps) than other diagnoses or more severe groups.

In the past, eligibility for financial or social support in Taiwan was based on either impairment or diagnosis, and the children with more severe levels of impairment or with more observable diagnoses such as motoric (e.g., cerebral palsy) or sensory deficits (e.g., hearing loss or visual impairments) were eligible for packaged services (especially pension or assistive technology or home services) matched to specific diagnoses and impairment levels.

Resource distribution, however, should be based on the consideration that children with social/emotional as well as less severe impairments also need tailored services (such as personal support and care)[11] aimed at the goal of optimal participation as framed by ICF-CY and consistent with age-appropriate milestones. The joint impairment-participation decision for the Disability Evaluation System, particularly for the large population of individuals with mild intellectual disability is now under construction in Taiwan [18]. The results of this study provide evidence to inform policy and decision making for the DES.

Overall, based on the profiles of independence and frequency among children with disabilities provided by Step 1 (Fig 2), restriction of independence in daily participation decreased with age whereas restriction of participation frequency varied across setting and diagnosis. These data suggest that frequency, defined as the performance dimension of participation, what children did in the past 6 months, is a more useful variable and context-dependent measure of participation than independence. The negative gap between independence-frequency became wider with age, especially in neighborhood and community settings and in children with mild impairment. Children with mild levels of impairments are probably more often in neighborhood and community settings and thus exposed to more complex and less adapted environments. A positive gap for participation was found for children with severe to profound levels of impairment in home and school settings especially children aged 6–9 years of age. It may be that families and schools provide more support to children of a younger age and with more severe impairment levels.

A group of children sharing the same diagnosis, such as CP, may show a wide range of disability/functioning in daily life and some may not have limitations in mobility. Children with various diagnoses may also share common limitations of functioning, especially for participation in specific settings because of the environmental support or barriers encountered.

Children’s participation provides valuable information beyond diagnoses for assessing children’s needs [46] in all education settings (classroom settings as well as school-related clinical settings) with implications for continuity in the transitions from one educational level to the next and into work and employment [47]. Further analysis of environmental factors other than physical settings is needed, such as parents’ perspective on the provision of opportunities for activities. Continued research on the social as well as physical factors of the environment is needed to identify the conditions and opportunities that can promote the participation of children with disabilities.

Several limitations of this study need to be considered. First, the distribution of children across levels of severity, diagnoses, and ages was not even. This resulted in varied mean scores as shown in Fig 3 especially for the children with CP and in a specific age range. Second, the comorbidity issue is an inevitable challenge in data analysis for the effects of diagnoses on functional outcomes. Therefore, we only performed statistical analysis for Step 2 and 3 where children with more than one type of diagnosis were excluded. Third, the FUNDES-Child was designed to obtain caregiver’s or parents’ perceptions of children’s independence and frequency of engaging in activities. Thus, all the information was based on ratings by caregivers or parents within the scope of the report format of the FUNDES-Child in Taiwan. Caregivers or parents may not be as familiar with children’s participation in the school setting as in the home. Self-rating of participation by children was not feasible in that children often would have difficulty comprehending the questions.

Parents rating for measuring children’ participation has been seen as a valid approach to explore the patterns of participation in different settings and ages [48,49]. For children capable of answering survey questions, they should respond for themselves [9] as the integration of the child’s perspective and choice is especially important for full participation in life as an active learner [32,50]. Future studies of FUNDES-Child will prioritize self-ratings of participation by children, perhaps using pictures of the activities. Fourth, the independence-frequency gap may be only one of several ways to conceptualize the role of the environment. While we believe this is one reasoned approach, others may emerge as these concepts develop over time. The relations between gaps and environmental factors need further studies.

### Conclusion

In conclusion, this large-sample study of participants drawn from a disability register system encompassing a broad range of disabilities, demographic characteristics, and ages provides a rather comprehensive picture of functional characteristics/limitations of children with developmental disabilities in Taiwan. Diagnoses, physical conditions and related severity of impairments and limitations of functioning and participation have been differentiated with the ICF/ICF-CY model [4,21] and validated with empirical data [5,17]. Findings for this study indicate that children’s participation depends not only on the severity of their impairments or diagnoses, but also on their age, the settings and supports provided by their environments. A priority for future research is thus further identification of environmental opportunities that positively impact frequency of participation in daily activities for children with developmental disabilities.
